# A Qualitative Exploration of the Lived Experience of Menopause Among Black Women in the United Kingdom

**DOI:** 10.1002/puh2.70168

**Published:** 2025-12-16

**Authors:** Rachael A. Charles, Tongai F. Chichaya

**Affiliations:** ^1^ Aston University, College of Health and Life Sciences Birmingham UK; ^2^ Coventry University, School of Health and Care Coventry UK

## Abstract

**Introduction:**

Despite increased research on menopause in the United Kingdom, there is a scarcity of research published on the experiences of Black women going through menopause. Studies suggest that culture, race and ethnicity influence how menopause is experienced. Evidence shows that Black women face racial health inequalities in the United Kingdom. Therefore, this study aimed to explore the menopause experiences of Black women in the United Kingdom.

**Methods:**

The interpretive phenomenological analysis (IPA) study design was used to explore Black women's lived experience of menopause in the United Kingdom. Purposive sampling was then used to select the five participants who were included in this study. In‐depth interviews were used for data collection and IPA was used for data analysis.

**Findings:**

Three main themes emerged from the study following data analysis. The themes are unprepared, coming to terms, and acceptance and cultural shift. The findings show that participants had less knowledge about menopause before they started experiencing the symptoms. Strategies used to cope with menopause include humour, dietary and lifestyle changes. A cultural shift towards viewing menopause as a transformative phase at mid‐life which should not to be stigmatised as a taboo subject was identified.

**Conclusion:**

Menopause is a challenging experience for Black women in the United Kingdom in many ways. These preliminary findings suggest the potential value of tailored approaches to symptom management.

## Graphical abstract

Three themes emerged: unprepared, coming to terms and acceptance with cultural shift. Participants reported limited knowledge of menopause, adopted coping strategies and highlighted a shift towards viewing menopause as a transformative, non‐stigmatised mid‐life phase which should not to be stigmatised as a taboo subject was identified.

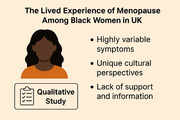

## 1 Introduction

Approximately 13 million people in the United Kingdom are experiencing peri/menopause, representing one‐third of the female population [1]. With Black people making up 4% of the UK's population [2], a significant number of Black women fall within this group. Menopause is when a woman's period stops for more than 12 consecutive months, marking the end of her reproductive life [3]. This gradual process is caused by reduced secretion of ovarian hormones, oestrogen and progesterone [4, 5]. Menopause is defined by three stages, perimenopause, menopause and post‐menopause [6], and typically occurs between 45 and 55 years of age [3]. Oestrogen receptors exist throughout the body, their decline leads to a wide range of symptoms, including vaginal changes, low libido, osteoporosis, brain fog, mood swings, sleep issues and hot flashes [7]. Prolonged symptoms can lower quality of life [8] and contribute to long‐term health risks like depression, anxiety and cardiovascular disease [9].

Individual experiences of menopause vary widely and are influenced by a range of personal and contextual factors [10, 11], including race, ethnicity, socio‐cultural background, socio‐economic status, religious beliefs, healthcare access and attitude towards ageing [12, 13, 14, 15]. Management of symptoms includes dietary and lifestyle changes, increased exercise and intake of vitamins and minerals to promote bone health and well‐being [16]. Hormone replacement therapy (HRT) is the standard medical approach used to restore oestrogen to the body to help manage symptoms [17].

There is scarcity of literature on menopause experiences among Black women in the United Kingdom with more studies focusing on White women. Only two UK‐based studies specifically focused on health issues for Black women. This could be partly due to recruitment biases that rely on algorithms targeting dominant demographics [18]. Available research often focuses on the US context. This research often highlighted vasomotor symptoms (VMS) as a predominant symptom experienced by Black women. However, a UK study of 84 Black women reported symptoms, including fatigue, tiredness, reduced libido and muscle aches as the main symptoms of menopause [19].

Blanken et al. [20] report that Black women have lower odds of reporting their symptoms. Rice [21] argues that underreporting of menopause symptoms may be attributed to racial health disparities and a lack of trust in healthcare systems. Literature shows that Black women in the United Kingdom experience racial health inequalities and significant issues with accessing healthcare, which affects how they manage their health issues [22, 23, 24, 25]. This further highlights the need for more research which investigates Black women's perspectives and experiences of menopause in the United Kingdom, which this study aimed to investigate.

A UK study by Ozuzu‐Nwaiwu [26] with 22 Black women found that many had limited understanding of menopause, with some reporting inadequate information from GPs, misdiagnosis and pressure to use HRT. This may reflect gaps in cultural sensitivity in healthcare services. In the United Kingdom, the recommended treatment for managing menopause symptoms is HRT [17], yet some evidence shows that Black women are less likely to be willing to use it [4, 19, 26]. Clinicians may lack awareness of race‐specific menopausal symptoms and risks [27]. More research on Black women's experiences could inform policies in healthcare, employment and social services, leading to more inclusive support for Black women during menopause. Therefore, this study aimed to explore the menopause experiences of Black women in the United Kingdom.

## 2 Reflexivity

The first author is a 40‐year‐old Black woman from London with a history of reproductive health issues, contributing to the passion for this research area. Her lived experience and anecdotal evidence showed that the topic of menopause among Black women in the United Kingdom is justifiably regarded as a private, sensitive matter. The first author conceptualised the research, conducted the study and drafted the original manuscript. Her position helped to establish a good rapport and develop trust with the participants, allowing them to feel comfortable discussing their personal experiences. This may not have been achievable for an outside researcher with whom the participant may not have identified. The second author, a Black man with expertise in qualitative research, acknowledges potential biases stemming from his lack of lived experience with menopause as a Black woman in the United Kingdom. He contributed guidance, expertise and editorial support to refine the manuscript and ensure the study's trustworthiness in preparation for publication. The article results from scholarly collaboration between the first author and the second author.

Both authors used bracketing to set aside preconceptions or personal opinions regarding the views of the participants and the data with an open mind [28, 29]. The first author used memoing to document their personal assumptions, emotional dispositions and positionality before commencing data collection. This process of bracketing was further strengthened through peer briefing with the second author. Reflective discussions were used to identify and address any potential bias before and during data analysis, as well as during the writing of the findings. During data analysis, interpretations of the data were directly linked to transcripts to ensure the participants' viewpoints were expressed without the researchers’ bias. This was achieved by using direct quotes from the participants to support each theme from this study.

## 3 Methodology

This study used the interpretive phenomenological analysis (IPA) study design and methodology to enable in‐depth exploration of Black women's perspectives and experiences of menopause in the United Kingdom. Interpretative phenomenological analysis enables unpacking emotionally complex reactions to specific times of change in people's lives [30]. Furthermore, IPA aims to understand participant experiences first‐hand, accepting them as part of the reality associated with the phenomenon under investigation [31]. The phenomenon of interest in this study was the menopause experiences of Black women in the United Kingdom. The ontological stance adopted in this study is relativism, which seeks to understand reality by exploring the perspectives and interpretations of the participants regarding their reality of experiencing menopause [32].

Ethics approval was obtained from the psychology research ethics committee at the University of Derby with ethics approval number ETH2223‐2075. Following ethics approval, participants were recruited online via Facebook social networking website. A poster with unrestricted viewership was used to advertise the study. Following permission from group moderators, the poster was also shared in Facebook groups related to Black women in the United Kingdom or menopause. Potential participants were screened via Qualtrics online survey, inclusion criteria included women over 18 years who experienced peri/menopause within the last 5 years in the United Kingdom and who racially identify as Black. The exclusion criteria included women who experienced menopause because of surgical treatment. The Qualtrics survey allowed the potential participants to express interest in participating in the study and to consent to being contacted for the purposes of the study. Purposive sampling was then used to select participants for inclusion in the study from those who had expressed interest.

Eleven responses were received, two participants did not provide consent, two provided consent but did not include contact information, one participant began her transition over 8 years ago, one participant lived outside of the United Kingdom whilst experiencing perimenopause. Five participants were included in the study after providing consent and getting clarification on the study's aims. The sample met IPA requirement for a homogenous sample [30]. It consisted of a similar demographic group where age, birthplace and menopausal stage were concerned. Table 1 shows the characteristics of the five participants included in the study.

**TABLE 1:** Participant characteristics.

Pseudonym	Age	Menopausal stage	Married	Children	Employed	Country of birth	Ethnicity
Femi	46	Perimenopause	Yes	Yes	Yes	United Kingdom	Black British African
Beverly	50	Perimenopause	Yes	No	Yes	United Kingdom	Black British African
Sophia	51	Perimenopause	No	No	Yes	United Kingdom	Black British African
Shirley	51	Menopause	No	Yes	Yes	United Kingdom	Black British Caribbean
Shazzi	52	Menopause	Yes	Yes	Yes	United Kingdom	Black British Caribbean

In‐depth interviews were conducted online and recorded using MS Teams. The interview schedule included topics related to participants’ experiences with menopause, awareness, symptom management and cultural perceptions. The first author conducted all interviews and gained verbal consent at the beginning and end of each. The interviews lasted between 40 and 75 min. Observational notes documented non‐verbal information such as expressions, gestures, body language and silence. The transcript for each interview was checked to ensure accuracy before being downloaded from MS Teams. These five participants were the only women who volunteered to be part of the study and who met the inclusion criteria.

Data analysis was conducted using IPA following the steps outlined by Smith and Nizza [33]. Step 1 involved working through transcripts line by line to note clusters of meaning. In Step 2, the notes were used to form experiential statements. In Step 3, experiential statements were used to create a structure to explain what happened in each case. Subthemes around the personal experiences of each participant were identified in Step 4. Step 5 involved conducting a cross‐case analysis to compare findings and establish the main themes.

## 4 Findings

The five participants were aged between 46 and 52 years old. They were all born in the United Kingdom and identified themselves as being of Black African or Black Caribbean descent. They lived in the United Kingdom when this study was conducted and had experienced menopause transition during the last 4 years. Table 1 shows the characteristics of the study participants. To maintain confidentiality and anonymity, pseudonyms were used to differentiate the participants in this study.

Following data analysis, three main themes emerged from the study: *unpreparedness*, *coming to terms*, and *acceptance and cultural shift*. Table 2 shows the respective sub‐themes for each main theme and the associated direct quotes from the participants.

**TABLE 2:** Main themes, sub‐themes and key quotes.

Main theme	Sub‐themes	Direct quotes from participants
Unprepared	Limited knowledge Uncertainty about symptoms Appearance of symptoms	*‘I didn't know it had stages. I thought it came, … before you're 50, … just appears. I didn't realise the havoc that it causes … whilst you're going through it’*. (Femi) *‘Nobody ever mentioned it, until I started going through it’*. (Femi) *‘The hot flushes for me were the first thing …’*. (Femi)
Coming to terms	Coping strategies Lifestyle adaptations Healthcare management support systems	*‘I am spending money in three different places just to get someone to advise me or support me’*. (Femi) *‘Can't do anything with hormones so you've got to try and manage’*. (Sophia)
Acceptance and cultural shift	Cultural views Gradual acceptance Self‐perception	*‘I'm not embarrassed by it. I don't think any less of myself for it’*. (Sophia)

## 5 Theme 1: Unprepared

Being unprepared was informed by factors such as having little knowledge about menopause before participants began to experience it. Participants’ understanding of menopause mainly stemmed from witnessing their older relatives experiencing symptoms like hot flushes and sweating. They perceive older generations as stoic and long‐suffering, having minimised the complexities associated with menopause. Having less knowledge resulted in participants underestimating the impact of menopause on daily life. All participants mentioned that their primary sources of information were friends, family and the media. Notably, health professionals or services were not cited as critical sources of information. This was interpreted to mean that participants trust friends, family and media more than health services.

Uncertainty about the onset of menopause contributed to experiencing unpreparedness. Three participants recognised changes in their menstrual cycle, best described by Shazzi as *‘on‐off periods* *’*, but they did not associate it with menopause. Shirley said, *‘It's when I was in full menopause that I … recognised what was going on’*. The presence of pre‐existing health conditions and medications used to treat symptoms contributed to two participants finding it particularly challenging to recognise the onset of menopause. For example, Femi said, *‘… when mine started I associated that with me having hyperthyroidism the symptoms were very similar’*.

Participants were unaware of the somatic changes associated with menopause and were unprepared for the experience. All five participants perceived themselves to be experiencing menopause when they experienced VMS, which seemed to be dramatic, as mentioned by Shazzi: *‘… every cell in your body has just turned on like a cooker’*. Two participants mentioned decreased libido, three experienced brain fog, two reported fatigue, anxiety and low mood. Irritability was a major concern for two participants; one participant sought help for depression. Three participants had sleep disturbances, two mentioned weight gain.

The psychological symptoms involved heightened irritability and extreme mood swings that are difficult to control and lingering, these were especially challenging. Beverly explained, ‘*… when you're in that mood, you understand how some people … cross that line to being suicidal’*. Femi described volatile, *‘yo‐yo’* moods. Three participants viewed themselves as difficult to be around and reported the psychological symptoms to be worse than physical ones due to their intensity and ability to derail thoughts, prompting them to question their mental state. Sophia, Femi and Shirley reported brain fog as unpredictable, affecting work performance and professional image. Sophia said, *‘My everyday speaking brain has … holes in it’*, whereas Femi stated, *‘I felt like I was losing the respect of my team’*.

## 6 Theme 2: Coming to Terms

Participants use short‐term adaptive behaviours to manage symptoms, such as lighter duvets, layering clothes and carrying face cloths. They describe a cycle of adjusting clothing, windows and fans throughout the day. Three participants used humour as a way of coming to terms with their experiences. For example, Shazzi jokingly referred to the hot flushes she experienced as her ‘*own personal summer*.’ Despite this, participants struggle to mask psychological symptoms, often coping by isolating themselves at home and work. The interpretation of this is that participants did not want to be misunderstood or attract attention to their menopause experiences. Working from home was mentioned as a preferable option where possible because it allowed participants to manage work and address menopause symptoms privately.

Participants described adapting their routines to better manage symptoms in the long term. Some described being more introverted and planning earlier social events to avoid negative impact on sleep, which was perceived to lead to low mood. Four participants found lifestyle changes, like dietary adjustments and increased exercise were effective for symptom management. Two participants shared about excluding alcohol, sugars and incorporating yams, fruits, vegetables, ginseng, ginger and drinking okra. Femi ascribes her improvement to extreme dietary changes.

All participants also reported managing their health by taking vitamins, supplements and minerals to improve bone health, mood, sleep and the immune system. These include ashwagandha, maca root, turmeric, vitamins C, D, E and magnesium. Notably, those on HRT also used these supplements, implying that HRT alone was not considered to be sufficient.

There was a shared belief that plants have the medicinal elements required to manage their symptoms, with fewer side effects than medication. Menopause was perceived as a natural process that can be overcome without medication. As Sophia says, *‘I've never wanted to rely on something for what my body could sort’*, emphasising self‐reliance over seeking medical interventions. Those who do not intend to use medication view it as futile, reporting their symptoms to doctors who focus on prescribing HRT, ‘*there's nothing symptom‐wise that… a doctor can sort’*. This reflects a perceived lack of information and guidance towards alternatives to HRT. There were sentiments that HRT is associated with cancer, most sought alternative remedies until symptoms became unbearable.

Femi had a pre‐existing condition that prevented her from using HRT and expressed her frustration in receiving fragmented support. *‘I am spending money in three different places just to get someone to advise me or support me’*. Two participants who use HRT felt well‐supported. They view HRT as offering a quick, effective solution to coping with VMS. In addition, Beverly explained her appreciation for being offered counselling for depressive symptoms. Participants also expressed a need for menopause guidance tailored to their unique experiences as Black women. Shirley found it easier to trust and understand a Black menopause specialist, highlighting the value of relatable healthcare. She adds that representation builds trust and comfort in healthcare interactions. ‘*I've been quite lucky …my…menopause specialist, …she's black so that helped… And she was also in her 50's’*.

Identifying and using support systems was another strategy used by participants in coming to terms with menopause. Primary support was received from friends, family and peer groups who shared their experiences, validated their experiences, provided a safe space to discuss menopause, and shared strategies for coping. At work, female colleagues were considered more supportive and capable of understanding the challenges than male colleagues, who they viewed as less empathetic to menopause‐related challenges.

## 7 Theme 3: Acceptance and Cultural Shift

Participants’ levels of acceptance towards menopause were varied, most embraced it as an important, inevitable life stage. Participants view menopause as an important life event that warrants more discussion. Femi refers to the question on knowledge about menopause as a *‘million‐dollar question’*, which signifies its importance and value. Participants reported a notable cultural shift in the United Kingdom providing a new narrative, where menopause is widely discussed and normalised. They attributed this to media coverage, advocacy, and celebrity endorsements, which contributed to the topic's de‐stigmatisation. Beverly notes an increased focus on women's health issues in public discussions, whereas Sophia views it as a topic gaining importance but believes that women's health still requires greater attention overall.

Some participants have used acceptance to gain and maintain a positive outlook. Beverly reframed menopause as a *‘quest’* she could conquer with mental resilience and remedies. Most participants were self‐assured and viewed symptoms as momentary inconveniences rather than reflections of their worth or femininity. Participants find the *‘mind over matter’* approach to be helpful. Some participants viewed menopause as representing a sense of ‘loss’ of youthfulness, reproduction, libido and a reminder of their mortality. This was exacerbated by the perception that menopause is widely viewed as a ‘negative event’ on women's calendars. Others maintained a stable sense of self, unaffected by age or physical changes, and regarded this phase of life as offering increased self‐awareness and the privilege of being one's true self. Despite being self‐conscious when experiencing VMS in public, most viewed menopause as a natural part of ageing rather than something diminishing their capabilities or self‐esteem.

A cultural change towards viewing menopause as a transformative phase at mid‐life, empowering women to take control of their experiences through awareness and acceptance, was evidenced. This differs from the perceptions about their older generations, who they considered stoic and long‐suffering, having minimised the complexities associated with menopause by keeping them secret. Sophia said: *‘I don't think my mum had … symptoms…she's quite stoic… apart from hot sweats. She never complained about anything … so I don't know …. I've never had a proper conversation with her about it …’. Similarly, Femi said: ‘I don't see myself … playing it down as much as what they've done’*.

The cultural shift was also accompanied by a determination to normalise conversations about menopause. There was a strong perception among the participants that menopause should be treated like menstruation, involving more education, awareness and guidance, specifically from their mothers. This view is evident in Femi's statement: *‘… never been anything where anybody said, “Oh, Femi, sit down. We need to talk to you. You know, like … they would do about your menstrual cycle”’*.

Despite the identified cultural shift, participants believed that the discriminatory emphasis on youthfulness and productivity persists in UK culture. This has been linked to feeling unsupported or stigmatised at work, especially by male colleagues or supervisors. Shirley said: *‘…when I have my review at the end of the year, it will be interesting if my boss says, … “I've noticed … your lack of focus,” …. That will be a down point, so I'll get … less of a pay rise, less of a bonus, but really, it's actually a medical thing…’*. Similarly, Femi said: *‘I felt like I was losing the respect of my team’*. Advocating for greater workplace support, participants expressed a desire for policies that recognise and accommodate menopause, encouraging a supportive environment rather than one that side‐lines or penalises affected women.

## 8 Discussion

The findings indicate that menopause is a challenging experience for Black women in the United Kingdom in many ways. Participants experienced various physical and psychological symptoms that impacted their daily lives. The most common symptoms experienced by the participants were VMS, weight gain, brain fog, low mood and irritability. This study aligns with other studies reporting that Black women experience intense, frequent VMS [21, 26]. Some previous studies suggest that Black women experience less psychosomatic symptoms [34, 35]. However, this study found that the psychological symptoms of menopause were considered to be worse than the physical ones. This could be linked to the concerns around how the psychological symptoms affected work performance and interpersonal relationships. These challenges can be compounded in workplaces where menopause is still considered a taboo subject [36]. Moreover, in this study, there was a view that men in the workplace were less capable of understanding the participants’ experiences of menopause and less likely to empathise with or support them.

Participants in this study were unprepared for menopause because it was never discussed by older generations of Black women, who they perceived to have been stoic and guilty of minimising their own experiences. Participants reported having less knowledge regarding menopause, implying the need for more information to be provided to Black women who may be at increased risk of accessing inaccurate information from their social networks. This is consistent with previous research that suggests Black women had less understanding of menopause prior to experiencing it [15, 21, 26, 37]. The findings from this study suggest that Black women view menopause as an important life event deserving more discussion. There was a reported need to break the stigma and boundaries that have been associated with menopause, with some suggesting discussing menopause with the younger generation of Black women and participating in more research. This cultural shift could be the foundation of adequate support being provided to Black women experiencing menopause in the United Kingdom.

Friends, families and social media communities were the primary sources of information and support reported in this study. Participants described this as a more reliable support system than the healthcare services available to them. This offers Black women a safe space that allows them to process their experiences and come to terms with menopause. This aligns with prior studies [15, 21, 26]. The data also suggest that Black women feel more comfortable discussing their symptoms with women of a similar age, who they assume have more knowledge about menopause and are therefore more capable of understanding the challenges they encounter. This finding was evident in their discussions about work, visits to healthcare practices and the level of detail they comfortably shared during the data collection process of this investigation. This implies there is a universal feminine identity that is apparent at mid‐life. The absence of health and social services as sources of information could imply the lack of trust in health services among Black women in the United Kingdom. This was consistent with broader research by MacLellan et al. [38], who highlighted how discrimination and poor communication contributed to poorer outcomes and mistreatment for minority ethnic women's experience of UK maternity services. Similarly, Paul et al. [39] found that when ethnic minority groups experience racism in healthcare services, they were more likely to refuse the COVID‐19 vaccine due to their distrust in the healthcare system. However, two participants who used HRT reported feeling supported by health services, especially if the menopause specialist was relatable, a Black woman.

The findings suggest that Black women prefer to address symptoms naturally by making dietary and lifestyle changes and increasing their intake of vitamins and supplements during mid‐life. They are less inclined to use HRT unless symptoms become unbearable. This aligns with previous research that investigated Black women in the United Kingdom [19, 26]. This indicates that the current healthcare available in the United Kingdom could be perceived as less effective among Black women because it offers a medical approach to symptom management, which is not a ‘natural’ approach. Some participants did not view the current NHS guidance that is available as adequate to address their specific concerns about menopause, as their major concerns were about psychological and physical symptoms. This highlights the need for additional interventions to mitigate the psychological symptoms that Black women experience during menopause.

In addition, the data imply that Black women consider themselves to be lucky if a healthcare professional takes the time to thoroughly explain health guidance in a way that addresses their specific concerns. These inferences further suggest that Black women experience less relevant healthcare services in the United Kingdom during menopause, which also aligns with earlier research which suggests the services Black women receive are less relevant and partially ineffective [19, 26]. More inclusive studies that investigate menopause in a racially diverse sample of women are needed to ensure that the healthcare advice that is offered in the United Kingdom considers Black women and other ethnic minorities.

## 9 Limitations

This study had some limitations: The literature is scarce about menopause among Black women in the United Kingdom. Only two previous studies addressing menopause specifically for Black women in the United Kingdom were found. The study's small sample size does not capture the perspectives of Black women from a wide range of socio‐economic backgrounds in the United Kingdom. Recruitment via Facebook may have introduced further bias, potentially excluding women who are less digitally engaged or do not use Facebook. The study looked at three women who had children and two who did not. Very little is known about the impact of menopause on motherhood identity in the United Kingdom, especially for women without children. The findings of this study suggest that this is a knowledge gap which could be addressed by future research. A further limitation of this study is that it excluded women who started experiencing menopause more than 5 years from the time of data collection; however, some studies suggest that Black women may experience symptoms longer. Although the findings suggest valuable insights, they are exploratory and cannot be generalised to all Black women in the United Kingdom due to the small, homogenous sample, further highlighting the need for more research with a larger, diverse population and robust study designs.

## 10 Conclusion

This study aimed to explore the perspectives and experiences of menopause for Black women in the United Kingdom; the themes unprepared, coming to terms, acceptance and cultural shift were identified. This study has provided insights into the perspectives and experiences of menopause among Black women in the United Kingdom. These findings suggest that a more diverse approach to symptom management could ensure Black women feel more supported by the healthcare services that are available in the United Kingdom. Future research using larger, more socio‐economically diverse samples could enhance the understanding of Black women's experiences of menopause in the United Kingdom. Researchers could consider using a mixed‐methods survey to complement the findings from this IPA study. This is important because it could lead to the design of more effective interventions and healthcare services that are truly inclusive to meet the needs of Black women who experience menopause in the United Kingdom.

## Author Contributions

Rachael A. Charles took primary responsibility for the study, including conceptualisation, study design, data collection, data analysis and preparation of the initial manuscript draft. Tongai F. Chichaya acted in an advisory capacity, contributing to refinement of the methodology and data analysis, and assisting with critical review and final revisions of the manuscript.

## Conflicts of Interest

The authors declare no conflicts of interest.

## Data Availability

The data that support the findings of this study are available from the corresponding author upon reasonable request.
